# p53 Immunohistochemistry Patterns Are Surrogate Biomarkers for *TP53* Mutations in Gastrointestinal Neuroendocrine Neoplasms

**DOI:** 10.1155/2021/2510195

**Published:** 2021-12-15

**Authors:** Junjie Li, Jing Wang, Dan Su, Xiu Nie, Yueping Liu, Lianghong Teng, Junyi Pang, Huanwen Wu, Zhiyong Liang

**Affiliations:** ^1^Department of Pathology, State Key Laboratory of Complex Severe and Rare Diseases, Molecular Pathology Research Center, Peking Union Medical College Hospital, Chinese Academy of Medical Science and Peking Union Medical College, Beijing 100730, China; ^2^Department of Pathology, Cancer Hospital of the University of Chinese Academy of Sciences (Zhejiang Cancer Hospital), Hangzhou, 310022 Zhejiang, China; ^3^Department of Pathology, Union Hospital, Tongji Medical College, Huazhong University of Science and Technology, Wuhan, 430022 Hubei, China; ^4^Department of Pathology, The Fourth Hospital of Hebei Medical University/Hebei Cancer Hospital, Shijiazhuang, 050011 Hebei, China; ^5^Department of Pathology, Xuanwu Hospital Capital Medical University, Beijing 100053, China

## Abstract

**Aims:**

The aim of this study was to establish p53 immunohistochemistry (IHC) patterns to predict *TP53* mutations in gastrointestinal neuroendocrine neoplasms (GI-NENs) and to determine whether p53 IHC patterns could be used for the differential diagnosis of neuroendocrine neoplasms.

**Methods:**

*TP53* gene sequencing and p53 IHC were performed on formalin-fixed paraffin-embedded (FFPE) tissue samples from 92 patients diagnosed with GI-NENs from five medical centers.

**Results:**

The cohort included 35 well-differentiated neuroendocrine tumors and 57 poorly differentiated neuroendocrine carcinomas. Gene sequencing revealed 38 wild-type *TP53* and 54 *TP53* mutations. p53 expression was interpreted as follows: pattern A, p53 was absent from all tumor cells; pattern B, scattered and weak p53 expression in 1-20% of tumor cells; and pattern C was subclassified as pattern C1: variable p53 staining intensity in 21-60% of tumor cells and tumor cell nests with focal strong positive p53 staining and pattern C2: strong p53 staining in more than 60% of tumor cells. p53 IHC patterns were evaluated as a binary classifier where pattern B predicted wild-type *TP53*, and patterns A and C predicted *TP53* mutations. The sensitivity, specificity, and overall accuracy of this binary classification to predict *TP53* status were 0.963, 0.868, and 0.924, respectively. p53 IHC patterns were also correlated with *TP53* mutation types. Most cases with pattern A harboured loss-of-function (LOF) mutations, whereas patterns B and C tended to indicate wild-type *TP53* and gain-of-function (GOF) mutations, respectively. Furthermore, most of the well-differentiated NETs showed pattern B, whereas pattern C2 was more common in poorly differentiated NECs. Finally, staining interpretation between different observers also yielded high reproducibility.

**Conclusions:**

p53 IHC patterns may be used as predictors of *TP53* gene mutations and therefore could be potential surrogate markers for *TP53* mutations in GI-NENs and could distinguish between well-differentiated NETs and poorly differentiated NECs.

## 1. Introduction

Gastrointestinal neuroendocrine neoplasms (GI-NENs) are a group of heterogeneous diseases that occur in various digestive organs including the esophagus, stomach, small intestine, colon, and rectum. In the fifth edition of the WHO Classification of Tumors of the Digestive System, GI-NENs are generally classified as well-differentiated neuroendocrine tumors (NETs) and poorly differentiated neuroendocrine carcinomas (NECs) [1]. Morphologically, tumor cells of NETs are arranged in organoid, tubular, or sheet-like structures and have round or oval nuclei with “salt-and-pepper” chromatin. On the basis of mitotic rate and Ki-67 index, the well-differentiated NETs can be further graded as G1, G2, and G3. The poorly differentiated NECs include small-cell NECs and large-cell NECs. Small-cell NEC tumor cells have enlarged dark-staining nuclei and scant cytoplasm, whereas large-cell NEC tumor cells are of large size and have pleomorphic nuclei showing evident nucleoli. Although there are distinct morphological differences between NETs and NECs, distinguishing NETs, especially NET G3, from NECs based on morphological changes alone remains difficult in some cases.

GI-NENs are characterized by a series of molecular changes. Well-differentiated NETs derived from different digestive organs may harbour different mutations; for example, *CDKN1B* mutations can be found in 8% of the small intestine NETs [2], whereas some studies indicated that colorectal NETs have *PARP4* (28.6%) and *ATM* (15%) mutations [3, 4]. In contrast, *TP53* gene mutations are common in poorly differentiated NECs (63%-89%) [5–7], but rarely observed in well-differentiated NETs (less than 1%) [2–4]. Therefore, *TP53* mutation status can be used as a marker in the differential diagnosis of NETs and NECs.

p53 is a 393 amino acid protein encoded by *TP53* gene. As an important transcription factor and tumor suppressor, p53 protein participates in many biological processes, such as cell cycle arrest, cell apoptosis, DNA repair, and genomic stability [8]. p53 protein includes several functional domains, of which the DNA-binding domain performs cell cycle arrest and apoptosis functions in response to DNA damage [9]. *TP53* is one of the most frequently mutated oncogenes that occurred at rates from 5% to 50% in almost every type of cancer [10]. Mutations in *TP53* usually change the p53 protein (mostly the DNA-binding domain of p53 protein), altering p53 protein expression patterns and resulting in a gain-of-function or loss-of-function effects [11–13]. Gain-of-function (GOF) effects of *TP53* gene, known as the acquisition of oncogenic activities, are usually induced by missense mutation and in-frame indels [14–16]. Loss-of-functions effects, which are caused by nonsense mutations, splicing mutations, and frameshift indels, showed a decrease in the wild-type activity of p53 [17–19].

Studies have confirmed that p53 protein expression patterns as measured by immunohistochemistry (IHC) can be utilized as surrogate markers for *TP53* mutations in cancers of various organs. In gynaecological cancers, p53 IHC is widely accepted as a marker for *TP53* mutation status [20, 21]. For example, in ovarian carcinoma, p53 IHC has been proven to be a reliable predictor of *TP53* mutation status and can be used as a critical diagnostic marker for high-grade serous carcinoma [20]. Furthermore, according to the National Comprehensive Cancer Network Guideline (version 1.2021), p53 IHC is recommended for molecular subtype evaluation in patients with endometrioid carcinoma as a surrogate marker of *TP53* mutation status. Although some researchers have studied the expression of p53 in GI-NENs [22–24], however, it has not yet been determined in GI-NENs whether the expression of p53 is a reliable predictor and surrogate marker for *TP53* mutations.

In the present study, we analysed the relationship between p53 IHC and the *TP53* mutation status and types to clarify whether there is a correlation between them and tried to establish the criteria for p53 IHC interpretation in GI-NENs, as well as to determine whether the p53 IHC patterns could distinguish between well-differentiated NETs and poorly differentiated NECs.

## 2. Materials and Methods

### 2.1. Patients and Tissues

This study was approved by the Ethics Committee of Peking Union Medical College Hospital (HS-1908, March 26, 2019) and conducted in accordance with the Declaration of Helsinki. Informed consent was waived because the data were anonymized according to the research protocol. The specimens were collected from five medical centers including Peking Union Medical College Hospital, Cancer Hospital of The University of Chinese Academy of Sciences, Wuhan Union Hospital, Hebei Cancer Hospital, and Xuanwu Hospital Capital Medical University.

Candidates were retrospectively selected from the institutional databases of the five centers by searching for surgical resection specimens with key words “neuroendocrine tumor/neoplasm,” “neuroendocrine carcinoma,” or “neuroendocrine differentiation” diagnosed from 2010 to 2019. The primary location was identified as the GI tract, including esophagus, stomach, small intestine, and colorectum. The H&E slides were reviewed by two pathologists for consensus diagnosis of NETs and NECs prior to inclusion based on the latest WHO classification of digestive system tumors [1]. Clinicopathological characteristics including age, gender, primary site, tumor size, and TNM stage, were recorded. We excluded cases younger than 18 years, cases treated with preoperative radiation or chemotherapy, and specimens that were insufficient (tumor cells in the tissue sections < 50%) for mutational analysis or from metastatic sites. Patients were also eliminated if histology identified the diagnosis other than NETs/NECs (e.g., mixed neuroendocrine-nonneuroendocrine neoplasms) or if patients had incomplete clinicopathological information.

### 2.2. DNA Extraction, Sequencing, and Bioinformatics Analysis

For sequencing, the TIANamp Genomic DNA kit (Tiangen Biotech, Beijing, China) was used according the manufacturer's instructions to extract DNA from FFPE samples of primary tumor tissues and matched normal tissues. Whole-exome sequencing (WES) was performed on the Geneplus company's molecular laboratory platform. DNA fragmentation was performed using an UCD-200 ultrasonicator (Diagenode, Seraing, Belgium). AMpure beads (Beckman, MA, USA) were then used for purification and size selection of DNA fragments, according to the manufacturer's instructions. A Qubit 2.0 Nanodrop 2000 spectrophotometer and Qubit 2.0 Fluorometer with Quanti-IT dsDNA HS Assay Kit (Thermo Fisher Scientific, MA, USA) were used to assess DNA purity and concentration. Library construction was then performed using a 53 M length capturing probe (Integrated DNA Technologies, IDT, IA, USA), and the Geneplus-2000 sequencing platform (Geneplus, Beijing, China) was used to perform paired-end whole exome sequencing (WES) of DNA (read length of 100 bp).

Quality control of the raw data was performed using fastp [25]. After which, the high-quality clean sequences were aligned to the human reference genome (hs37d5(phase2), accessed in July, 2020) and sorted by BWA [26] and SAMtools [27]. The average sequencing depth was 371.30×. The average coverage, 30 × coverage, and 100 × coverage were 99.85%, 99.14%, and 88.28%, respectively. Then, the sequence file was processed by GATK [28], including marking the PCR duplicated reads by MarkDuplicates and recalibrating base quality scores by BaseRecalibrator and ApplyBQSR. The mutation calling was performed by MuTect2 to detect somatic SNVs and small indels. The parameters of these bioinformatics tools are summarized in Supplementary table S1.

After mutation calling, several procedures were designed for variant filtering. Firstly, high-frequency variants in unrelated individuals were filtered out as previously reported [29]. Then, we used the methods demonstrated in [30] to screen out the cancer associated nonsynonymous mutations. For those nonmissense mutations, candidate alterations that were cancer-associated genes characterized in the COSMIC, OncoKB, Civic, CGI, or IntOGEN databases (all accessed in July, 2020) were reserved. The missense mutations were determined by a FATHMM-MKL score of more than 0.5 in COSMIC database, or identified by two or more of the following criteria: (i) predication score 0-0.05 in SIFT, (ii) “possibly damaging” or “probably damaging” in Polyphen2, and (iii) prediction score > 0.5 in FATHMM-MKL. For these nonsynonymous mutations, missense mutations and in-frame indels were classified as GOF mutations, and nonsense mutations, frameshift indels, and splicing mutations were classified as LOF mutations.

### 2.3. p53 Immunohistochemistry

Fresh tissue samples were fixed with formalin and embedded in paraffin. The tissue block was then cut into 4 *μ*m sections, attached to a glass slide, and incubated at 60°C for 2 h. IHC staining was performed on the Ventana Benchmark Ultra platform (Roche, Tuscon, AZ, USA) according to standard operating procedures. A primary p53 monoclonal antibody (DO-7, ZSGB-Bio, Beijing, China) was used for staining.

Following IHC staining, the slides were evaluated by two pathologists who were blind to the results from the WES analysis. The cell counting was performed manually on screen using whole slide images of the IHC slides. Cells with signal in the nucleus were considered p53 positive, and staining of the mesenchyme, lymphocytes, and surrounding normal epithelium was used as the internal control. The percentage of positive cells per total tumor cells was calculated, and the staining intensity was recorded as weak, strong, or variable.

### 2.4. Statistical Analysis

IBM SPSS 22.0 (SPSS Inc., Chicago, Illinois, USA) was used for statistical analysis. All findings were considered statistically significant when *p* < 0.05. Fisher's exact test was used to evaluate the relationship between p53 staining patterns and *TP53* mutation status or the histological type of GI-NEN. The sensitivity, specificity, and overall accuracy were also calculated. The kappa agreement test and Pearson correlation analysis were used to assess the correlation of p53 IHC interpretation between different observers.

## 3. Results

### 3.1. Patient Population

We have collected 102 cases at the beginning, and 10 cases were dropped from the study because of unsuccessful DNA extraction and IHC staining. Finally, a total of 92 GI-NEN samples were used for subsequent analysis. The age of all patients at initial diagnosis ranged from 32 to 86 years old (median age 60.5). Fifty-seven (57/92, 62.0%) of the patients were men and 35 (35/92, 38.0%) were women. Lesions originated in the esophagus (7/92, 7.6%), stomach (50/92, 54.3%), small intestine (7/92, 7.6%), and colorectum (28/92, 30.4%). Forty-three (46.7%) patients had positive regional or distant metastasis at the time of primary diagnosis. Of these 92 GI-NEN cases, thirty-five (38.0%) cases were well-differentiated NETs, and the remaining 57 (62.0%) cases were poorly differentiated NECs. The detailed clinical data of NETs and NECs are summarized in Table 1.

### 3.2. Analysis of TP53 Mutations

Among the 92 GI-NEN cases, nonsynonymous *TP53* mutations were detected in 54 cases, including 37 cases with GOF mutations and 17 cases with LOF mutations, and the remaining 38 cases were wild type (Supplementary table S2). *TP53* mutations were detected in the majority of NECs (53/57, 93.0%), and only one mutation was observed in NET (1/35, 2.9%).

**Figure 1:**
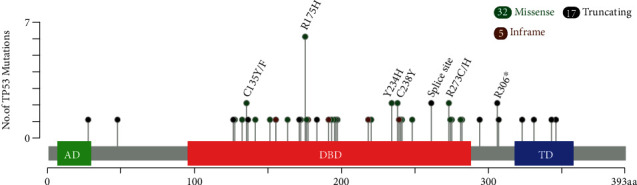
The “lollipop” plot generated by the MutationMapper tool of cBioPortal [31, 32] shows the open box of *TP53* gene, as well as the frequency and position of *TP53* mutations in GI-NENs in our study. AD: transactivation domain; DBD: DNA binding domain; MD: tetramerization domain.

Of the GOF *TP53* mutations in NECs, there were 32 (86.5%) missense mutations and five (13.5%) in-frame indels, and LOF mutations included eight (47.1%) nonsense mutations, four (23.5%) frameshift indels, and five (29.4%) splicing mutations. The remaining mutation in NET G3 was missense mutation. Most mutations occurred in exon 5-8 (44/54, 81.5%), and the most frequently mutated exon was exon 5 (20/44, 40.8%). Most of the mutations (44/54, 81.5%) were located in the DNA-binding domain (Figure 1). The most common amino acid substitutions were R175H (6/54, 11.1%).

### 3.3. p53 Immunohistochemistry

In order to establish the cut-offs of p53 IHC, we summarized the number of cases and the percentage of p53 positive cells stratified by an interval of 10% in 92 GI-NEN cases (Supplementary table S3). By the comparison between the percentage intervals and the results of *TP53* mutational analysis, we determined three cut-offs as 1%, 20%, and 60% for further IHC interpretation.

**Figure 2:**
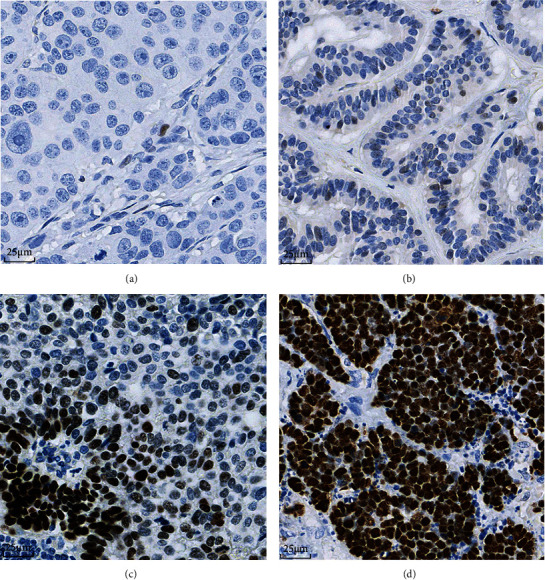
Different patterns of p53 expression in GI-NENs. (a) Pattern A showed completely absent p53 expression of tumor cell nucleus. Note that scattered stromal cells were weakly positive for p53 (40x). (b) Pattern B showed p53 expression in 1%-20% of tumor cell nuclei with weak intensity (40x). (c) Pattern C1 showed a variable staining intensity with focal tumor cell nests demonstrating strong positivity (40x). (d) Pattern C2 was accompanied by diffuse (61%-100%) and strong nuclear staining (40x).

Using the above cut-offs, three distinct immunohistochemical patterns of stained nuclei were identified based on both the percentage of positive cells and the staining intensity: pattern A (Figure 2(a)), tumor cells showed a complete absence of nuclear p53 immunolabeling with scattered weakly positive stromal cells serving as an internal reference for IHC assessment; pattern B (Figure 2(b)), 1-20% of tumor cells showed weakly positive staining in a scattered pattern; Pattern C, 21-100% tumor cells stained positive for p53. Then, cases with pattern C were classified as two subgroups according to the staining intensity: Pattern C1 (Figure 2(c)), 21-60% of tumor cells stained positive for p53 with variable intensity, and focal tumor cell nests with strong positive staining were present; pattern C2 (Figure 2(d)): 61-100% of tumor cells had diffuse and strongly positive p53 staining. The most common IHC pattern was pattern C (44/92, 47.8%, pattern C1:6/44, 13.6%, pattern C2: 38/44, 86.4%), followed by patterns B (35/92, 38.0%) and A (13/92, 14.1%).

### 3.4. Correlation between p53 IHC Patterns and TP53 Mutation Status

Overall, there was a significant correlation between p53 IHC staining patterns and *TP53* mutation status in GI-NENs (*p* < 0.001). By comparing the pattern types and mutation status, pattern B was more commonly observed (33/35, 94.3%) in wild-type cases than *TP53*-mutant cases, whereas pattern A (11/13, 84.6%) and pattern C (41/44, 93.2%) were more frequent in *TP53*-mutant cases. To implement a binary classification system to predict *TP53* mutation status, we categorized pattern B as wild-type pattern and pattern A and pattern C as mutant pattern. The sensitivity, specificity, and overall accuracy of this binary IHC pattern classification approach for *TP53* mutation status prediction were 0.963 (95% CI 0.862–0.994), 0.868 (95% CI 0.711–0.951), and 0.924 (95% CI 0.849-0.965), respectively (Table 2).

### 3.5. Correlation between p53 IHC Patterns and TP53 Mutation Types

We then analysed the relationship between p53 IHC patterns and *TP53* mutation types (Table 3). Most of the cases with IHC staining pattern A showed LOF mutations (11/13, 84.6%). In these LOF mutations with pattern A, nonsense mutations were the most common and found in five cases (45.5%), followed by splicing mutations (3/11, 27.3%) and frameshift indels (3/11, 27.3%). Thirty-three cases with wild-type *TP53* showed pattern B (94.3%), and *TP53* mutations were detected in only two of the 35 pattern B cases. 84.1% (37/44) of cases with pattern C showed GOF mutations. Among the six cases with IHC staining pattern C1, half (3/6, 50.0%) harboured missense mutations, and the remaining cases were nonsense, splicing mutations, and in-frame indel. Of the 38 cases with IHC staining pattern C2, missense mutations were the most common and detected in 29 cases (76.3%), followed by in-frame indels (4/38, 10.5%), wild type (3/38, 7.9%), nonsense mutation (1/38, 2.6%), and frameshift indel (1/38, 2.6%).

### 3.6. p53 IHC Patterns in NETs and NECs

**Figure 3:**
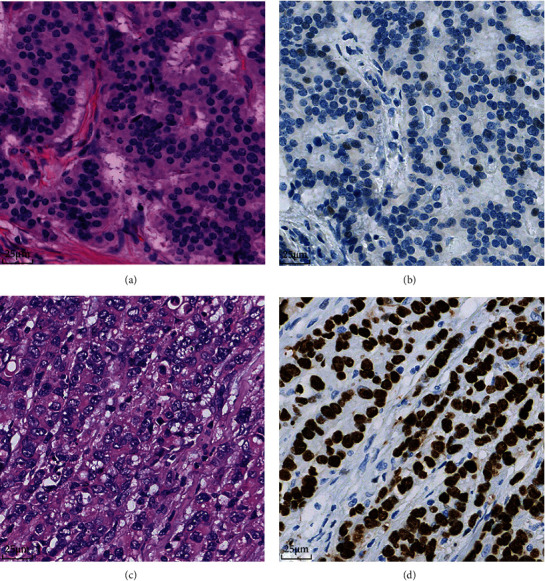
The relationship between histological subtypes and p53 IHC pattern in GI-NENs. (a, b) A case of well-differentiated NET with wild-type *TP53* and pattern B of p53 expression (40x). (c, d) A poorly differentiated NEC case with *TP53* missense mutation and pattern C2 (40x).

As summarized in Figure 3 and Table 4, most of the well-differentiated NETs showed pattern B (31/35, 88.6%), followed by pattern A and pattern C2. No pattern C1 was observed in NET cases. Among 57 poorly differentiated NECs, pattern C2 was the most common staining pattern (36/57, 63.2%) followed by pattern A and pattern C1. Only 7.0% (4/57) of the poorly differentiated NECs showed pattern B.

### 3.7. Reproducibility of the p53 IHC Patterns

**Figure 4:**
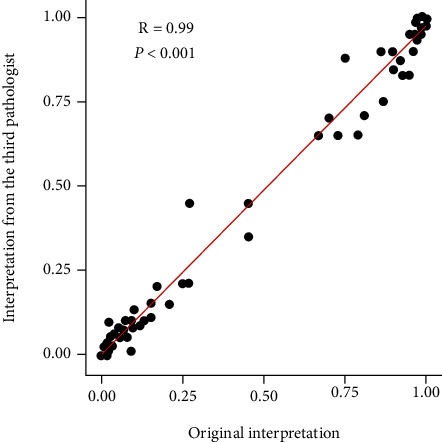
The Pearson analysis showed a strong correlation between the percentages of p53 immunoreactive cells by different pathologists (*R* = 0.99, *p* < 0.001).

In order to test the reproducibility of the established patterns of p53 IHC expression, we invited the third pathologist to assess the p53 slides of all GI-NENs independently and recorded the patterns as well as the percentage of p53-positive tumor cells. The result of interpretation indicated that there existed a good concordance among different pathologists (Supplementary table S4, kappa = 0.967, *p* < 0.001), and the p53-positive percentages produced by the pathologists were also found highly concordant (Figure 4). These results indicated that the p53 IHC patterns we established were highly reproducible between different pathologists.

## 4. Discussion

*TP53* mutations are common in tumors from various organs, and p53 IHC is an established surrogate marker for *TP53* mutations in gynaecological tumors. However, the relationship between p53 IHC and *TP53* mutation has not been fully explored for GI-NENs. *TP53* mutation status is valuable for the differential diagnosis of GI-NENs [33], but detecting *TP53* mutation requires sequence analysis, which is time-consuming and labour-intensive. p53 IHC is a straightforward method that is routinely used in histopathological diagnosis, which makes it an easy and fast alternative approach to assessing *TP53* mutation status. Therefore, it is important to determine whether p53 IHC can predict *TP53* mutations in GI-NENs.

Previous researches have shown that NETs and NECs are correlated with different molecular alterations, and *TP53* mutations are more common in NECs than NETs [1, 5, 6]. In our study, the majority of NECs had *TP53* mutations, whereas *TP53* mutations were exceedingly rare in NETs. In agreement with previous studies [33, 34], most *TP53* mutations were located in exon 5-8, and more than half of the *TP53*-mutant cases in our cohort exhibited missense mutations. In addition, our study showed that *TP53* mutations occurred most frequently in DNA-binding domain in GI-NENs.

It has been proved that abnormal p53 protein expression usually presents an “all or nothing” pattern in many cancers [20]. However, there is no consensus regarding the cut-offs of p53 IHC interpretation to infer *TP53* mutations in GI-NENs. The cut-offs from early studies were either empirical without *TP53* mutations as references, or only based on high-grade NENs from various sites [7, 24]. Different from the previous works, in the present study, three p53 staining patterns (patterns A, B, and C) were identified based on the percentage of p53-positive tumor cells and the p53 staining intensity in GI-NENs with reference to the results of *TP53* mutations. Pattern A corresponded to the complete absence of p53 expression, pattern B denoted wild-type expression, and pattern C indicated overexpression. Intriguingly, we identified a unique p53 staining pattern in pattern C as pattern C1, which was characterized by variable p53 staining and focal tumor cell nests with strong staining intensities. Although variable staining intensity was usually considered an indicator of wild-type *TP53* [21, 24, 35], however, in the present study, pattern C1 was considered to be a mutant staining pattern as most of cases with pattern C1 showed *TP53* mutations. Furthermore, we found that p53 staining patterns were closely correlated with *TP53* mutation status (wild-type versus mutant) and further categorized these three patterns as wild-type (pattern B) and mutant (pattern A and C). Our binary IHC classification demonstrated both high sensitivity and specificity for predicting *TP53* mutation status, which indicates that p53 IHC can be a reliable tool for determining *TP53* mutation status.

Notably, in cases with pattern A, complete absence of p53 IHC staining in tumor cells could be confirmed only when the internal controls (stromal cells, lymphocytes, and adjacent normal epithelial cells) showed scattered weakly positive p53 staining [24]. In a small number of cases, p53 expression was completely absent throughout the entire section including both tumor cells and nontumor internal controls. The IHC results from these cases were considered uninterpretable, and this may have occurred due to poor fixation and preservation. In these cases, repeating IHC staining or direct gene sequencing on the same or alternative blocks is warranted for *TP53* mutation prediction or detection. In the present study, we excluded two uninterpretable cases that showed completely absent p53 expression in both tumor and internal control cells but harboured wild-type *TP53* or missense mutations (Supplementary figure S1).

We also found that p53 staining patterns were associated with *TP53* mutation types. *TP53*-mutant cases with patterns A and C most frequently harboured LOF and GOF mutations, respectively. Similar findings have been observed in gynaecological tumors [20, 21]. Recent work has begun to clarify the mechanisms by which abnormal p53 expression patterns can predict the presence and types of mutations in *TP53*. IHC staining of p53 protein in tumor cells is dependent on its expression and degradation. Under normal circumstances, wild-type p53 undergoes rapid conformational changes that result in its degradation; therefore, only weak and scattered p53 expression is detected by IHC. However, GOF mutations in p53 can prolong its half-life, which leads to diffuse and strong IHC staining [36], and LOF mutations may cause truncation of the p53 protein, which results in completely negative p53 staining [37].

Although p53 staining has been known to be associated with *TP53* gene mutations, however, there are also some studies indicating that p53 staining cannot distinguish between different mutations of *TP53* gene, especially between wild-type and nonsense mutations [38–42]. The following two reasons may potentially explain this. First, different IHC evaluation methods may result in inconsistent results between different studies. For example, Schoop et al. used three algorithms to evaluate p53 IHC staining, but completely negative pattern and scattered weakly positive pattern were considered a single staining category [41]. As a result, wild-type *TP53* and nonsense mutations cannot be discriminated in this study. Second, nonsense mutations located in the 3′-end of the *TP53* gene may result in protein truncation without affecting the p53 antibody binding site. Hence, cases with *TP53* nonsense mutations may have wild-type p53 staining.

It is worth noting that although our samples were collected from multiple research centers, the sample size in our study was nonetheless limited. Therefore, further validation is needed to determine the importance of p53 IHC staining patterns, especially pattern C1, in GI-NENs. Another limitation of this study is the absence of an additional cohort for external validation. It is well recognized that the use of an external dataset can reduce the bias and validate the robustness of the model in clinical research [43]. This limitation may be addressed by the efforts in data sharing with other clinical organizations in the future. In summary, our results revealed that p53 IHC staining patterns in GI-NENs are associated with *TP53* mutation status and types. p53 IHC patterns may be a feasible method for *TP53* mutation prediction in view of the high availability of this technology and could distinguish between well-differentiated NETs and poorly differentiated NECs as well.

## Figures and Tables

**Table 1:** Clinicopathological characteristics of 92 GI-NENs.

Clinicopathological features	NET no. (%)	NEC no. (%)
Sex		
Male	18 (51.4)	39 (68.4)
Female	17 (48.6)	18 (31.6)
Age at diagnosis		
Median	53	66
Mean	50.9	65.0
Range	32-72	47-86
Tumor location		
Esophagus	1 (2.9)	6 (10.5)
Stomach	12 (34.2)	38 (66.7)
Small intestine	3 (8.6)	4 (7.0)
Colorectum	19 (54.3)	9 (15.8)
TNM stage		
I-II	26 (74.3)	30 (52.6)
III	5 (14.3)	18 (31.6)
IV	4 (11.4)	9 (15.8)
Regional/distant metastasis		
Positive	5 (14.3)	38 (66.7)
Negative	30 (85.7)	19 (33.3)

**Table 2:** Comparison between p53 IHC staining patterns and *TP53* mutation status in 92 GI-NEN cases.

IHC staining patterns	Mutation status		*p* value
	Wild-type	Mutant type	
Pattern types			
Pattern A	2	11	<0.001
Pattern B	33	2	
Pattern C	3	41	
Binary classification			
Wild-type pattern	33	2	<0.001
Mutant pattern	5	52	

**Table 3:** Comparison between p53 IHC patterns and *TP53* mutation types in 92 GI-NEN cases.

IHC staining pattern	Mutation types					
	LOF			Wild type	GOF	
	Nonsense	Frameshift	Splicing		Missense	In-frame
Pattern A	5	3	3	2	0	0
Pattern B	1	0	1	33	0	0
Pattern C1	1	0	1	0	3	1
Pattern C2	1	1	0	3	29	4
Total	8	4	5	38	32	5

**Table 4:** Comparison of p53 IHC staining patterns among NET and NEC cases.

IHC patterns	NETs, no. (%)	NECs, no. (%)	*p* value
Pattern A	2 (5.7)	11 (19.3)	<0.001
Pattern B	31 (88.6)	4 (7.0)	
Pattern C1	0	6 (10.5)	
Pattern C2	2 (5.7)	36 (63.2)	

## Data Availability

All data included in this study are available upon request by contact with the corresponding author.

## Supplementary Materials

Supplementary Materials Supplementary table S1: the optional parameters of the tools in the bioinformatics analysis. Supplementary table S2: the distribution of *TP53* status in 92 GI-NENs. Supplementary table S3: comparison between the percentage of p53 positive cells and *TP53* mutation status in 92 GI-NEN cases. Supplementary table S4: the inter-pathologist concordance for p53 IHC patterns in GI-NENs. Supplementary figure S1: the examples of uninterpretable cases. p53 expression was completely absent throughout the entire section including both tumor cells and nontumor internal control cells (40x) in one case with wild-type *TP53* (A) and another case with *TP53* missense mutation (B).
