# Supplementary material for: p53 Immunohistochemistry Patterns Are Surrogate Biomarkers for TP53 Mutations in Gastrointestinal Neuroendocrine Neoplasms
Source: Gastroenterol Res Pract. 2021 Dec 15;2021:2510195. doi: 10.1155/2021/2510195 (PMC8695011; doi:10.1155/2021/2510195)
Supplement: Supplementary Materials — Supplementary table S1: the optional parameters of the tools in the bioinformatics analysis. Supplementary table S2: the distribution of TP53 status in 92 GI-NENs. Supplementary table S3: comparison between the percentage of p53 positive cells and TP53 mutation status in 92 GI-NEN cases. Supplementary table S4: the inter-pathologist concordance for p53 IHC patterns in GI-NENs. Supplementary figure S1: the examples of uninterpretable cases. p53 expression was completely absent throughout the entire section including both tumor cells and nontumor internal control cells (40x) in one case with wild-type TP53 (A) and another case with TP53 missense mutation (B). [file 2510195.f1.pdf]

Supplementary table S1 The optional parameters of the tools in the bioinformatics analysis

| Tools | Optional parameters |
|---|---|
| **fastp (v0.20.0)** | -f 10 -t 0 -F 10 -T 0 -w 8 --length_required 35 -q 15 -u 40 -n 5 -3 1 |
| **BWA mem2(v2.1)** | mem -Y -M -R "@RG\tID:"···"\tSM:"···"\tLB:"···"\tPU:"···"\tPL:"ILLUMINA"" |
| **SAMtools (v1.9)** | sort --reference hs37d5.fa |
| **MarkDuplicates<br>(packed in GATK 4.1.2.0)** | --VALIDATION_STRINGENCY SILENT<br>--OPTICAL_DUPLICATE_PIXEL_DISTANCE 2500<br>--ASSUME_SORT_ORDER "coordinate"<br>--CLEAR_DT false<br>--CREATE_MD5_FILE true |
| **BaseRecalibrator<br>(packed in GATK 4.1.2.0)** | -R hs37d5.fa<br>--use-original-qualities<br>--known-sites Mills_and_1000G_gold_standard.indels.b37.vcf<br>--known-sites dbsnp_138.b37.del100.vcf.gz<br>--known-sites 1000G_phase1.indels.b37.vcf.gz |
| **ApplyBQSR<br>(packed in GATK 4.1.2.0)** | -R hs37d5.fa<br>--add-output-sam-program-record<br>--use-original-qualities |
| **Mutect2<br>(packed in GATK 4.1.2.0)** | --germline-resource af-only-gnomad.raw.sites.b37.vcf.gz<br>--af-of-alleles-not-in-resource 0.0000025<br>-A ClippingRankSumTest |

Supplementary table S2 The distribution of *TP53* status in 92 GI-NENs

| ***TP53 Mutations*** | | **NET** | **NECs** |
|---|---|---|---|
| **LOF** | **Nonsense** | 0 | 8 |
| | **Frameshift indels** | 0 | 4 |
| | **Splicing** | 0 | 5 |
| **Wild type** | | 34 | 4 |
| **GOF** | **Missense** | 1 | 31 |
| | **In-frame indels** | 0 | 5 |

Supplementary table S3 Comparison between the percentage of p53 positive cells and *TP53* mutation status in 92 GI-NEN cases

| **Percentage of p53 positive cells (%)** | **Mutational Analysis** | | | | | | **Total** |
|---|---|---|---|---|---|---|---|
| | **LOF** | | | **Wild Type** | **GOF** | | |
| | **Nonsense** | **Frameshift** | **Splicing** | | **Missense** | **In-Frame** | |
| **0** | **5** | **3** | **3** | 2 | 0 | 0 | 13 |
| **1-10** | 1 | 0 | 1 | **28** | 0 | 0 | 30 |
| **11-20** | 0 | 0 | 0 | **5** | 0 | 0 | 5 |
| **21-30** | 1 | 0 | 1 | 0 | **2** | **0** | 4 |
| **31-40** | 0 | 0 | 0 | 0 | **0** | **0** | 0 |
| **41-50** | 0 | 0 | 0 | 0 | **1** | **1** | 2 |
| **51-60** | 0 | 0 | 0 | 0 | **0** | **0** | 0 |
| **61-70** | 0 | 1 | 0 | 1 | **1** | **0** | 3 |
| **71-80** | 0 | 0 | 0 | 1 | **2** | **0** | 3 |
| **81-90** | 0 | 0 | 0 | 1 | **3** | **2** | 6 |
| **91-100** | 1 | 0 | 0 | 0 | **23** | **2** | 26 |

Supplementary table S4 The inter-pathologist concordance for p53 IHC patterns in GI-NENs

| **Original interpretation** | **Interpretation from the third pathologist** | | | |
|---|---|---|---|---|
| | **Pattern A** | **Pattern B** | **Pattern C1** | **Pattern C2** |
| **Pattern A** | 13 | 0 | 0 | 0 |
| **Pattern B** | 1 | 34 | 0 | 0 |
| **Pattern C1** | 0 | 1 | 5 | 0 |
| **Pattern C2** | 0 | 0 | 0 | 38 |

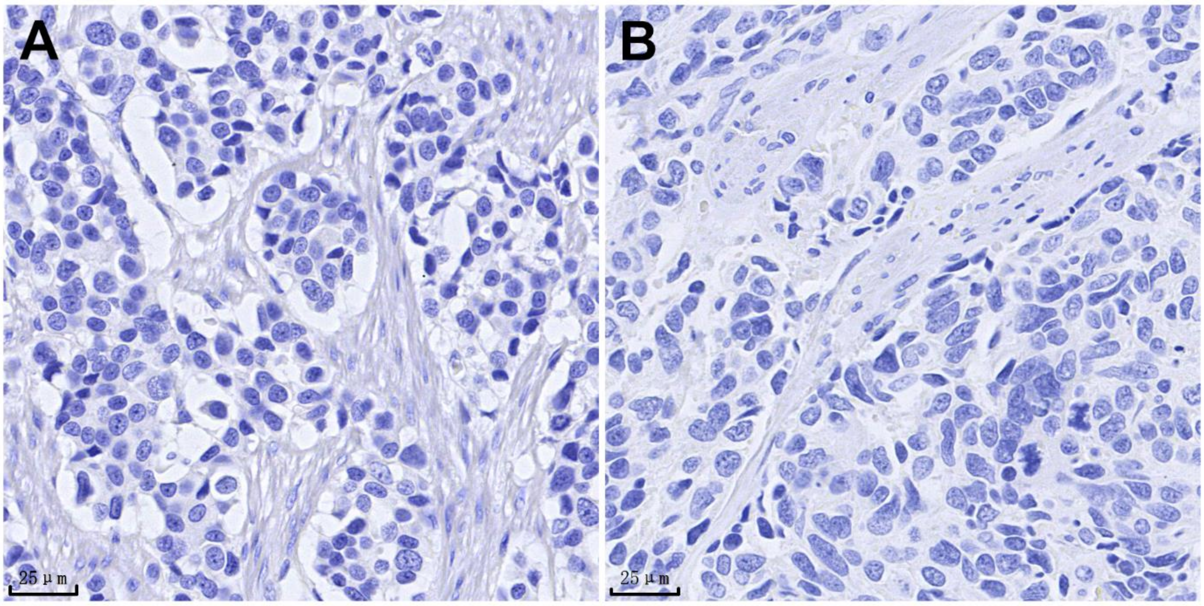


Supplementary figure S1. The examples of uninterpretable cases. p53 expression was completely absent throughout the entire section including both tumor cells and non-tumor internal control cells (40×) in one case with wild-type *TP53* (A) and another case with *TP53* missense mutation (B).
